# Application of an artificial intelligence-based tool in [^18^F]FDG PET/CT for the assessment of bone marrow involvement in multiple myeloma

**DOI:** 10.1007/s00259-023-06339-5

**Published:** 2023-07-26

**Authors:** Christos Sachpekidis, Olof Enqvist, Johannes Ulén, Annette Kopp-Schneider, Leyun Pan, Anna Jauch, Marina Hajiyianni, Lukas John, Niels Weinhold, Sandra Sauer, Hartmut Goldschmidt, Lars Edenbrandt, Antonia Dimitrakopoulou-Strauss

**Affiliations:** 1 Clinical Cooperation Unit Nuclear Medicine, German Cancer Research Center (DKFZ), Im Neuenheimer Feld 280, 69210 Heidelberg, Germany; 2 Eigenvision AB, Malmö, Sweden; 3 Department of Electrical Engineering, Chalmers University of Technology, Gothenburg, Sweden; 4 Division of Biostatistics, German Cancer Research Center (DKFZ), Heidelberg, Germany; 5 Institute of Human Genetics, University of Heidelberg, Heidelberg, Germany; 6 Department of Internal Medicine V, University Hospital Heidelberg and National Center for Tumor Diseases (NCT), Heidelberg, Germany; 7 Department of Clinical Physiology, Region Västra Götaland, Sahlgrenska University Hospital, Gothenburg, Sweden; 8 Department of Molecular and Clinical Medicine, Institute of Medicine, Sahlgrenska Academy, University of Gothenburg, Gothenburg, Sweden

**Keywords:** Multiple myeloma, [^18^F]FDG PET/CT, Deep learning, Artificial intelligence, Metabolic tumor volume (MTV), Total lesion glycolysis (TLG), Objective quantification

## Abstract

**Purpose:**

[^18^F]FDG PET/CT is an imaging modality of high performance in multiple myeloma (MM). Nevertheless, the inter-observer reproducibility in PET/CT scan interpretation may be hampered by the different patterns of bone marrow (BM) infiltration in the disease. Although many approaches have been recently developed to address the issue of standardization, none can yet be considered a standard method in the interpretation of PET/CT. We herein aim to validate a novel three-dimensional deep learning-based tool on PET/CT images for automated assessment of the intensity of BM metabolism in MM patients.

**Materials and methods:**

Whole-body [^18^F]FDG PET/CT scans of 35 consecutive, previously untreated MM patients were studied. All patients were investigated in the context of an open-label, multicenter, randomized, active-controlled, phase 3 trial (GMMG-HD7). Qualitative (visual) analysis classified the PET/CT scans into three groups based on the presence and number of focal [^18^F]FDG-avid lesions as well as the degree of diffuse [^18^F]FDG uptake in the BM. The proposed automated method for BM metabolism assessment is based on an initial CT-based segmentation of the skeleton, its transfer to the SUV PET images, the subsequent application of different SUV thresholds, and refinement of the resulting regions using postprocessing. In the present analysis, six different SUV thresholds (Approaches 1–6) were applied for the definition of pathological tracer uptake in the skeleton [Approach 1: liver SUV_median_ × 1.1 (axial skeleton), gluteal muscles SUV_median_ × 4 (extremities). Approach 2: liver SUV_median_ × 1.5 (axial skeleton), gluteal muscles SUV_median_ × 4 (extremities). Approach 3: liver SUV_median_ × 2 (axial skeleton), gluteal muscles SUV_median_ × 4 (extremities). Approach 4: ≥ 2.5. Approach 5: ≥ 2.5 (axial skeleton), ≥ 2.0 (extremities). Approach 6: SUV_max_ liver]. Using the resulting masks, subsequent calculations of the whole-body metabolic tumor volume (MTV) and total lesion glycolysis (TLG) in each patient were performed. A correlation analysis was performed between the automated PET values and the results of the visual PET/CT analysis as well as the histopathological, cytogenetical, and clinical data of the patients.

**Results:**

BM segmentation and calculation of MTV and TLG after the application of the deep learning tool were feasible in all patients. A significant positive correlation (*p* < 0.05) was observed between the results of the visual analysis of the PET/CT scans for the three patient groups and the MTV and TLG values after the employment of all six [^18^F]FDG uptake thresholds. In addition, there were significant differences between the three patient groups with regard to their MTV and TLG values for all applied thresholds of pathological tracer uptake. Furthermore, we could demonstrate a significant, moderate, positive correlation of BM plasma cell infiltration and plasma levels of β2-microglobulin with the automated quantitative PET/CT parameters MTV and TLG after utilization of Approaches 1, 2, 4, and 5.

**Conclusions:**

The automated, volumetric, whole-body PET/CT assessment of the BM metabolic activity in MM is feasible with the herein applied method and correlates with clinically relevant parameters in the disease. This methodology offers a potentially reliable tool in the direction of optimization and standardization of PET/CT interpretation in MM. Based on the present promising findings, the deep learning-based approach will be further evaluated in future prospective studies with larger patient cohorts.

## Introduction

[^18^F]FDG PET/CT is an imaging modality of high performance in the management of patients with multiple myeloma (MM) [1–4]). Foremost, due to its ability to reliably differentiate metabolically active from inactive lesions, [^18^F]FDG PET/CT is considered the appropriate method for treatment response evaluation in the disease [3, 4]. On the other hand, [^18^F]FDG PET/CT carries some limitations in MM evaluation. Some of these limitations are rather general, derived from the non-specific nature of the tracer, such as several false-positive findings [5], while some are more specific for MM, including a non-negligible (11%) incidence of false-negative results [1, 6]. Moreover, one particular challenge that clinical specialists and radiologists commonly face in MM is the standardization of the evaluation of PET/CT scans. This issue is mainly due to the different patterns of bone marrow (BM) infiltration in the disease, which, in turn, may hamper inter-observer reproducibility in interpreting scan results [7].

In recent years, many different approaches have been developed in order to address the issue of standardization of [^18^F]FDG PET/CT evaluation in MM. These approaches have made use of visual [7, 8] as well as semi-quantitative and quantitative [1–12] approaches. Although all these attempts seem promising, none can yet be considered a standard and widely accepted method in the interpretation of PET/CT. In this context, visual evaluation of the PET/CT scans remains the mainstay in clinical routine.

Deep learning, a subfield of artificial intelligence (AI), has nowadays become the method of choice for automated image analysis [13]. This method provides new opportunities for the development of automated analysis tools for CT, PET/CT, and MRI, which have the potential to improve or replace current methods for the evaluation of these imaging modalities [14]. Still, although the number of studies in this field is constantly growing, a large body of the literature is dominated by retrospective cohort studies with limited external validation and a high probability of bias [15–19]. Particularly with regard to MM, there are no data on the application of deep learning tools for the assessment of malignancy using PET/CT.

Accordingly, the aim of this prospective study is to evaluate a novel three-dimensional deep learning-based tool on PET/CT images for automated assessment of the intensity of BM metabolism in MM patients.

## Materials and methods

### Patients

Thirty-five consecutive patients (26 male, 9 female; mean age 59.3 years) with previously untreated MM based on the criteria established by the International Myeloma Working Group (2014) were included in this analysis (Table 1) [20]. All patients were investigated in the context of an open-label, multicenter, randomized, active-controlled, phase 3 trial (GMMG-HD7) [21]. No patient had previously received chemotherapy, granulocyte colony-stimulating factor (G-CSF), or erythropoietin. All patients gave written informed consent after the study was fully explained to them. The study was conducted in accordance with the International Conference on Harmonization Good Clinical Practice guidelines and the Declaration of Helsinki principles, and with institutional approval by the ethical committee of the University of Heidelberg (AFmu-412/2018) and the Federal Agency of Radiation Protection in Germany (“Bundesamt für Strahlenschutz”).

**Table 1:** Patient characteristics (*N* = 35)

Patient characteristics	Value
Median age, years	62 (41–65)
Sex	
Male	26 (74%)
Female	9 (26%)
Median hemoglobin, g/dL	12.3 (7.7–15.9)
Median albumin, g/dL	42.0 (25.1–54.3)
Median β2-microglobulin, mg/L	2.7 (1.0–13.6)
Median LDH, u/L	195 (70–420)
Free light chain ratio (κ/λ)	6.5 (0.1–1925.3)
Median bone marrow plasma cell infiltration	38% (4–100%)
High-risk cytogenetics†	
Yes	8 (23%)
No	23 (66%)
Unknown	4 (11%)
ISS	
1	25 (71%)
2	3 (9%)
3	7 (20%)
R-ISS	
1	17 (49%)
2	11 (31%)
3	3 (9%)
Not defined	4 (11%)

†High-risk cytogenetics defined as the presence of at least one of the following mutations: del(17)(p13), t(4;14)(p16;q32), or t(14;16)(q32;q23)

### PET/CT data acquisition

All patients underwent whole-body [^18^F]FDG PET/CT at diagnosis before commencement of treatment including induction therapy, high-dose chemotherapy (HDT), and autologous stem cell transplantation (ASCT), as well as maintenance therapy based on lenalidomide. All patients were normoglycemic at the time of the PET study. Imaging was performed at a mean time of 64 min (range: 51–79 min) postinjection (p.i.) of [^18^F]FDG from the skull to the toes with an image duration of 2 min per bed position. A dedicated PET/CT system (Biograph mCT, S128, Siemens Co., Erlangen, Germany) with an axial field of view of 21.6 cm with TruePoint and TrueV, operated in a three-dimensional mode, was used. A low-dose attenuation CT (120 kV, 30 eff mA) was used for attenuation correction of the PET data and for image fusion. All PET images were attenuation-corrected, and an image matrix of 400 × 400 pixels was used for iterative image reconstruction. Iterative image reconstruction was based on the ordered subset expectation maximization (OSEM) algorithm with two iterations and 21 subsets as well as time of flight (TOF).

### PET/CT data analysis

[^18^F]FDG PET/CT images were analyzed on an Aycan workstation. Two experienced, board-certified nuclear medicine physicians well versed in MM diagnosis (with 11 and 30 years of clinical experience in MM PET diagnostics—first and last authors, respectively) read and interpreted the datasets in consensus. The qualitative analysis was based on the visual assessment of the PET/CT scans. In particular, BM/skeletal foci presenting with enhanced (higher than background bone) [^18^F]FDG uptake, for which another benign etiology—for example, trauma or arthritis—was excluded, were considered positive for myeloma. These foci were correlated with the fused low-dose CT findings to ensure higher diagnostic accuracy. Nevertheless, even foci of increased [^18^F]FDG uptake without corresponding osteolysis on CT were accounted for as MM-positive, since in general terms, metabolic processes take place earlier than morphological changes [22]. The number of [^18^F]FDG-avid lesions was calculated in each patient since this parameter is of prognostic significance in newly diagnosed MM, with a higher number of lesions being associated with adverse progression-free survival (PFS) and overall survival (OS) [23, 24].

Moreover, the degree of diffuse [^18^F]FDG uptake in the BM was estimated both visually, mainly employing the maximum intensity projection (MIP) PET images, and semi-quantitatively, after the calculation of the standardized uptake value (SUV) in the iliac bone and the lower lumbar spine. Due to its reasonably uniform tracer uptake, the liver parenchyma was used for background measurements by positioning the spheric VOIs in the right liver lobe, if without lesions, and at least 1 cm away from the edge of the liver. Based on these, the uptake in the BM was classified as negative/mild (< liver uptake), moderate (> 1.1 × liver uptake), and intense (> 2 × liver uptake) [7].

Furthermore, patients were classified into three groups based on the combination of the aforementioned findings and similar approaches applied in the literature in the field [8, 25]: group A, including patients with no focal lesions and negative/mild diffuse BM uptake (< liver). Group B, including patients with 1–3 focal lesions and/or moderate diffuse BM uptake (> liver uptake, + 10%). Group C, including patients with > 3 focal lesions and/or intense diffuse BM uptake (> > liver uptake, twice).

### Automated quantification method

The proposed deep learning-based method consists of the following three steps:

1. CT-based organ segmentation
2. Application of SUV threshold(s) inside the relevant organs
3. Refinement of the resulting regions using postprocessing

The convolutional neural network (CNN) described in [14] was used to segment 17 different bones as well as the liver and the gluteus maximus muscle. The bones were divided into *bones of the axial skeleton*, including the vertebrae, scapulae, clavicles, sternum, ribs, sacrum, and pelvic bones, and into *bones of the extremities*, including the humeri, ulnae, radii, hands, femora, patellae, tibiae, fibulae, tali, and feet. The skull was excluded frοm the evaluations to avoid the effect of the intense physiological [^18^F]FDG uptake from the brain.

The CT-based segmentation was transferred to the SUV PET images, and, subsequently, different SUV thresholds were applied to identify BM infiltration. All pixels with SUV above or equal to the threshold were segmented as positive for infiltration by MM. In the present analysis, six different SUV thresholds were applied for the definition of pathological tracer uptake in the skeleton:

- *Approach 1*: For the bones of the axial skeleton, a threshold of (liver SUV_median_) × 1.1 was used. Respectively, for the bones of the extremities, a threshold of (gluteus maximus SUV_median_) × 4 was applied.
- *Approach 2*: For the bones of the axial skeleton, a threshold of (liver SUV_median_) × 1.5 was used, and for the bones of the extremities, a threshold of (gluteus maximus SUV_median_) × 4.
- *Approach 3*: For the bones of the axial skeleton, a threshold of (liver SUV_median_) × 2 was used, and for the bones of the extremities, a threshold of (gluteus maximus SUV_median_) × 4.
- *Approach 4*: Every bone was given a threshold of ≥ 2.5 according to Terao et al. [12].
- *Approach 5*: For the bones of the axial skeleton, a threshold of ≥ 2.5 was used, and for the bones of the extremities, a threshold of ≥ 2.0 was used.
- *Approach 6*: The SUV_max_ in the liver was used for all bones as proposed in the respective literature [7, 8, 26] (Table 2).

**Table 2:** The different SUV thresholds applied for the definition of pathological tracer uptake in the BM with the AI tool, their median values (range) in the studied cohort, and the respective *r* and *p* values of the correlation analysis between MTV, TLG, and the biopsy-derived BM infiltration rate by malignant plasma cells

AI-approaches	Applied threshold	Median MTV (range), mL	Median TLG (range), g	Correlation with BM infiltration rate	Correlation with β2-microglobulin
Approach 1	*Axial skeleton*: liver SUV_median_ × 1.1 *Extremities*: gluteal muscles SUV_median_ × 4	134.9 (2.1–3569.7)	429.7 (5.4–12500.9)	*r* = 0.42, *p* = 0.01 (MTV)* *r* = 0.39, *p* = 0.02 (TLG)*	*r* = 0.39, *p* = 0.02 (MTV)* *r* = 0.37, *p* = 0.03 (TLG)*
Approach 2	*Axial skeleton*: liver SUV_median_ × 1.5 *Extremities*: gluteal muscles SUV_median_ × 4	8.6 (0–2769.3)	35.0 (0–11121.1)	*r* = 0.37, *p* = 0.03 (MTV)* *r* = 0.35, *p* = 0.04 (TLG)*	*r* = 0.33, *p* = 0.05 (MTV) *r* = 0.35, *p* = 0.04 (TLG)*
Approach 3	*Axial skeleton*: liver SUV_median_ × 2 *Extremities:* gluteal muscles SUV_median_ × 4	2.6 (0–2128.7)	9.5 (0–9629.8)	*r* = 0.26, *p* = 0.13 (MTV) *r* = 0.23, *p* = 0.18 (TLG)	*r* = 0.28, *p* = 0.11 (MTV) *r* = 0.28, *p* = 0.10 (TLG)
Approach 4	2.5	60.5 (0–2164.8)	164.9 (0–9793.5)	*r* = 0.35, *p* = 0.04 (MTV)* *r* = 0.35, *p* = 0.04 (TLG)*	*r* = 0.35, *p* = 0.04 (MTV)* *r* = 0.35, *p* = 0.04 (TLG)*
Approach 5	*Axial skeleton*: 2.5 *Extremities*: 2.0	60.5 (0–2204.7)	164.9 (0–9887.7)	*r* = 0.36, *p* = 0.04 (MTV)* *r* = 0.36, *p* = 0.04 (TLG)*	*r* = 0.36, *p* = 0.04 (MTV)* *r* = 0.35, *p* = 0.04 (TLG)*
Approach 6	SUV_max_ liver	0 (0–1561.9)	0 (0–8038.0)	*r* = 0.19, *p* = 0.29 (MTV) *r* = 0.18, *p* = 0.30 (TLG)	*r* = 0.07, *p* = 0.70 (MTV) *r* = 0.07, *p* = 0.69 (TLG)

*Statistically significant correlation (*p* < 0.05)

The reason for the use of SUV_median_ in Approaches 1–3 lies in the fact that median values are more robust to outlier SUV values, e.g., due to small inconsistencies in organ segmentation or PET/CT misalignment.

Due to the poor resolution of PET images, tracer uptake from adjacent tissue might spill over into the bone mask. Therefore, in order to alleviate this effect, the following steps were employed:

1. Each pixel in the mask was assigned to its local maximum using Meyer’s flooding algorithm [27].
2. Each pixel in the mask was then given the same label as its local maxima.
3. All connected components (18-connectivity) with volumes less than 1 mL were removed.

**Fig. 1:**
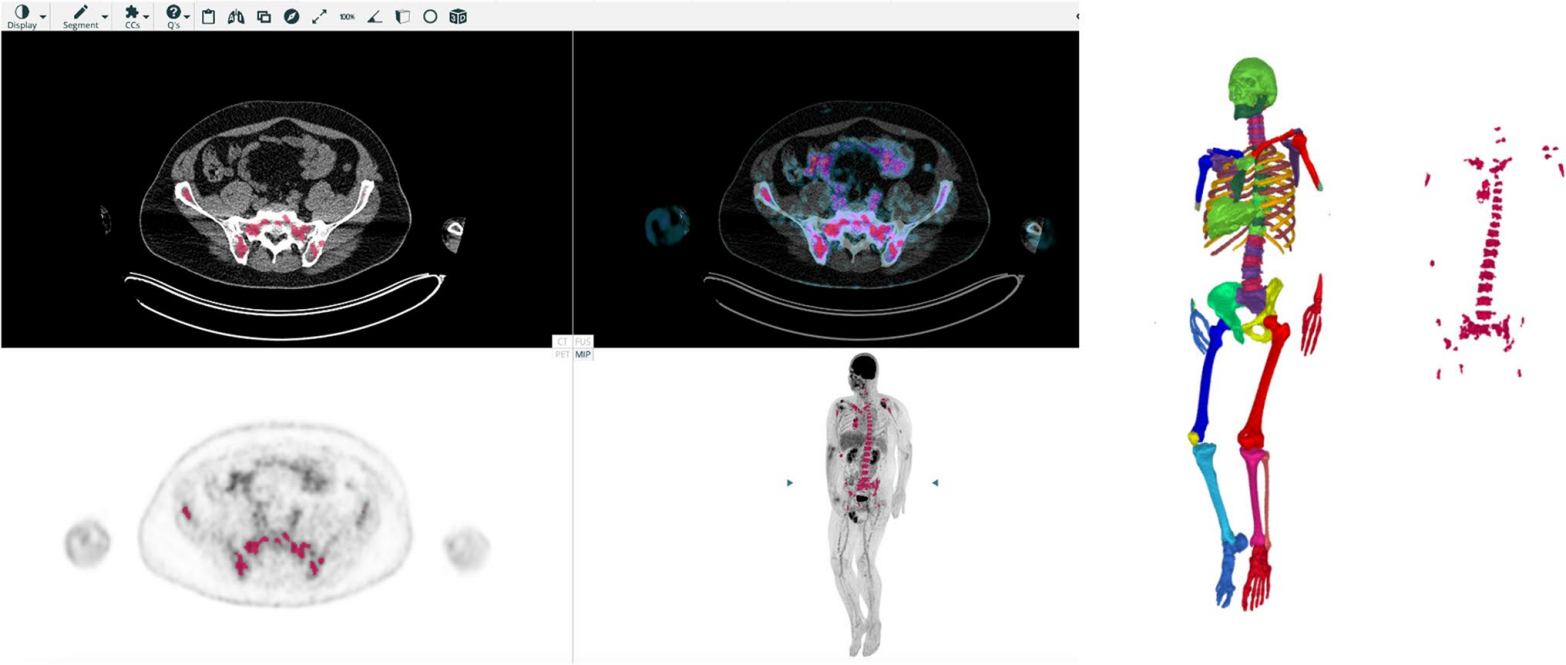
Image processing methodology for calculation of whole-body MTV and TLG with the application of the deep learning-based software tool. Myeloma lesions in the bone marrow compartment (red) are visualized on a standard display of PET/CT (left). The lesions are calculated based on an AI-based bone segmentation in CT illustrated in the 3D image (second from the right) and appropriate SUV thresholds for each group of bones resulting in bone lesion segmentations illustrated in the 3D image to the right. The resulting lesion segmentation (red) can be used to compute the total MTV and TLG for the patient

Using the resulting masks, the total, whole-body metabolic tumor volume (MTV) could be estimated as the volume of the segmented high uptake in each patient. In particular, MTV (mL) represents the myeloma lesions’ volume visualized on PET/CT with SUV greater than a pre-defined threshold (absolute value or relative to other organs). Similarly, the total lesion glycolysis (TLG) was estimated as the product of the average SUV and MTV for the segmented regions (TLG = SUV_mean_ × MTV) (Fig. 1).

### Clinical parameters, BM plasma cell infiltration, and fluorescence in situ hybridization

Thirty-four patients received BM biopsies from the iliac crest performed within 4 weeks of the [^18^F]FDG PET/CT examination and prior to the commencement of treatment. BM trephines were analyzed using hematoxylin–eosin stain, periodic acid–Schiff stain, and Giemsa stain. The percentage of BM infiltration by plasma cells was assessed via a light microscope. The infiltration rate represents the number of plasma cell in comparison to all nucleated cells in the BM. The monoclonality of plasma cells was confirmed by immunohistochemical staining.

Cytogenetic analyses were performed on CD138-purified BM plasma cells. High-risk cytogenetics was defined as the presence of at least one of the following aberrations (cutoff, ≥ 10% of cells): del(17)(p13), t(4;14)(p16;q32), or t(14;16)(q32;q23) (21). For the definition of high-risk disease, the Revised International Staging System (R-ISS) score was defined. Based on this prognostic system, stage R-ISS I included patients of ISS stage I (serum β2-microglobulin level < 3.5 mg/L and serum albumin level ≥ 3.5 g/dL), no high-risk chromosomal abnormalities, and a normal lactate dehydrogenase (LDH) level; stage R-ISS III included ISS stage III (serum β2-microglobulin level > 5.5 mg/L) and high-risk chromosomal abnormalities or a high LDH level; stage R-ISS II included all the other possible combinations [28].

### Statistical analysis

For all approaches, MTV and TLG measurements showed a skewed distribution. Therefore, median and range values are reported. Consequently, the correlation analysis of MTV and TLG measurements with BM infiltration rate and β2-microglobulin was based on Spearman’s rank correlation. For two-group comparisons (high-risk versus standard-risk cytogenetic abnormalities), the Wilcoxon rank sum test was used. To investigate whether there is a positive or negative trend in MTV and TLG measurements with ISS stage, R-ISS-stage, or groups A, B, and C from PET visual analysis, the (non-parametric) Jonckheere-Terpstra test for trend was used from the R package DescTools. The Jonckheere-Terpstra test was used for the investigation of the trend between the results of the PET visual analysis and the BM infiltration rate. The receiver operating characteristic (ROC) curve was used to investigate the performance of MTV and TLG for discrimination of the population according to the degree of BM infiltration rate, based on the BM plasma cell cut-off of 60% (≥ 60% versus < 60%). The area under the curve (AUC) was calculated, and the cut point optimizing the sum of sensitivity and specificity was determined for each Approach. Calculations were performed with R version 4.1.1 with packages DescTools and pROC. *p* values below 0.05 were considered statistically significant.

## Results

### Patient cohort

The plasma cell infiltration, as derived from BM biopsies, ranged between 4 and 100%, with a mean value of 42% (median = 38%). Cytogenetic data were available in 31 patients (89%), with high-risk cytogenetic abnormalities being detected in 8/31 (26%) of them. A combination of the ISS and cytogenetic data was available in 31 patients. Based on this, 17 patients were classified in the R-ISS-1 group (55%), 11 patients in the R-ISS-2 group (35%), and three patients in the R-ISS-3 group (10%). The patients’ characteristics are summarized in Table 1.

### [18F]FDG PET/CT findings

#### Visual analysis

Based on the results of the visual (qualitative) analysis of the PET/CT scans, 12 patients were classified into group A, 8 patients into group B, and 15 patients into group C. No statistically significant trend was observed between the results of the PET/CT visual analysis and the BM plasma cell infiltration. There were no cases of marked misalignment between PET and CT due to patient movement. Moreover, no patient exhibited an increased gluteal muscle uptake of reactive origin.

#### Automated quantification method

**Fig. 2:**
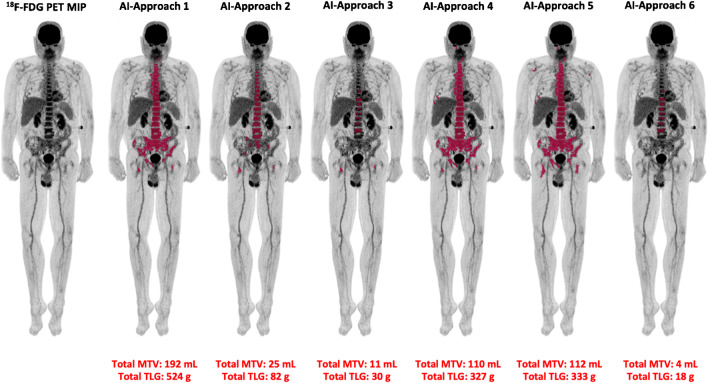
Example of the application of the AI-based software tool for automated calculation of total MTV and TLG of a MM patient with intense diffuse BM [^18^F]FDG uptake, therefore visually classified in group C. The use of different tracer uptake thresholds leads to different BM segmentation patterns and, subsequently, to different MTV and TLG values

**Fig. 3:**
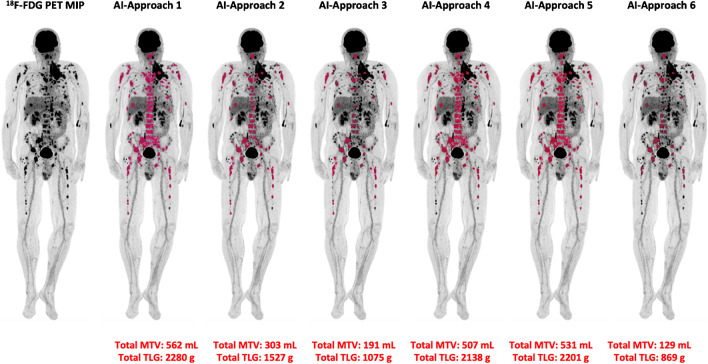
Example of the application of the AI-based software tool for automated calculation of total MTV and TLG of a MM patient with multiple focal [^18^F]FDG-avid lesions, therefore visually classified in group C. The use of different tracer uptake thresholds leads to different BM segmentation patterns and, subsequently, to different MTV and TLG values. Of note is the presence of a large myeloma bone lesion originating from the second left rib and infiltrating the adjacent soft tissues, of which only the osseous and not the paramedullary part is identified and segmented. Accurate calculation of paramedullary disease (PMD) represents a potential challenge for the implementation of the software tool

Six different SUV thresholds were applied for the definition of pathological tracer uptake in the skeleton and the subsequent AI-based, automated calculation of the MTV and TLG values. The results of this analysis are presented in Table 2. Examples of the application of the AI-based software tool for automated calculation of whole-body MTV and TLG in two MM patients are presented in Figs. 2 and 3.

### Correlation between automated quantitative PET/CT parameters and visual PET/CT evaluation

A significant (*p* < 0.001) positive trend was observed between the results of the visual analysis of the PET/CT scans, based on the classification of patients in groups A, B, and C, and the MTV and TLG values after the application of all six [^18^F]FDG uptake thresholds. In addition, there were significant differences between the three patient groups with regard to their MTV and TLG values for all applied thresholds.

### Correlation between automated quantitative PET/CT parameters, BM plasma cell infiltration, and clinical data

Exploratory correlation analysis revealed a significant, moderate, positive correlation between the automated quantitative PET/CT parameters, MTV and TLG, and BM plasma cell infiltration as well as plasma levels of β2-microglobulin after the utilization of the thresholds applied in Approaches 1, 2, 4, and 5 (Table 2). On the other hand, no significant correlations were observed for these parameters when employing Approaches 3 and 6.

In an attempt to evaluate the performance of the automated tool in providing information on myeloma disease severity, we dichotomized the population based on the cut-off of BM plasma cells of 60% and investigated the performance of whole-body MTV and TLG for the discrimination of patients based on this histopathological feature by ROC analysis. In line with the previous, Approaches 1, 2, 4, and 5 provided the best results regarding discrimination of population according to the degree of BM plasma cell infiltration, as reflected by the respective AUC being significantly different from 0.5. Cut points optimizing the sum of sensitivity and specificity were identified. The detailed results of the ROC analysis are presented in Tables 3 and 4.

**Table 3:** Area under the curve (AUC), 95% confidence interval (95% CI) of AUC, *p* values for testing whether AUC = 0.5, MTV thresholds optimizing the sum of sensitivity and specificity, as well as sensitivity, specificity, and accuracy at this threshold according to the different approaches for the discrimination of patient population based on the degree of bone marrow plasma cell infiltration (≥ 60% versus < 60%)

AI-approaches	AUC	95% CI	*p*	MTV threshold, mL	Sensitivity	Specificity	Accuracy
Approach 1	0.822	0.616–1	< 0.01*	443.4	0.78	0.92	0.88
Approach 2	0.789	0.579–0.999	0.01*	54.7	0.78	0.88	0.85
Approach 3	0.716	0.474–0.957	0.05	71.2	0.67	0.92	0.85
Approach 4	0.769	0.549–0.989	0.02*	267.4	0.67	0.92	0.85
Approach 5	0.771	0.547–0.995	0.02*	292.7	0.67	0.92	0.85
Approach 6	0.68	0.468–0.892	0.07	7.5	0.55	0.92	0.79

*Statistically significant results

*AUC*, area under the curve; *95% CI*, 95% confidence interval; *MTV*, metabolic tumor volume

**Table 4:** Area under the curve (AUC), 95% confidence interval (95% CI) of AUC, *p* values for testing whether AUC = 0.5, TLG thresholds optimizing the sum of sensitivity and specificity, as well as sensitivity, specificity, and accuracy at this threshold according to the different approaches for the discrimination of patient population based on the degree of bone marrow plasma cell infiltration (≥ 60% versus < 60%)

AI-approaches	AUC	95% CI	*p*	TLG threshold, g	Sensitivity	Specificity	Accuracy
Approach 1	0.804	0.603–1	< 0.01*	985.8	0.78	0.88	0.85
Approach 2	0.776	0.567–0.984	0.02*	146.6	0.78	0.84	0.82
Approach 3	0.698	0.46–0.936	0.07	132.0	0.67	0.88	0.82
Approach 4	0.764	0.545–0.984	0.02*	799.2	0.78	0.88	0.82
Approach 5	0.767	0.543–0.99	0.02*	856.1	0.67	0.92	0.85
Approach 6	0.676	0.464–0.887	0.07	34.0	0.56	0.92	0.79

*Statistically significant results

*AUC*, area under the curve; *95% CI*, 95% confidence interval; *TLG*, total lesion glycolysis

In contrast, no significant correlation was observed with the ISS-stage and the R-ISS stage for any of the applied thresholds. Moreover, no statistically significant differences were found between patients with high-risk abnormalities and those with standard cytogenetic risk, regarding automated PET parameters.

## Discussion

The interpretation of [^18^F]FDG PET/CT in MM may prove particularly challenging since both focal and diffuse bone lesions may coexist with varying degrees of [^18^F]FDG uptake. In clinical routine, the evaluation of BM involvement is primarily visual and subjective in nature, with quantitative—thus more objective—assessments being mainly restricted in the calculation of the semi-quantitative parameter, SUV, which is, however, susceptible to several factors, such as the reconstruction and acquisition parameters, partial-volume correction, blood glucose, and time between [^18^F]FDG injection and image acquisition [25], which affect its reliable and reproducible measurement. However, the standardized and reproducible interpretation of [^18^F]FDG PET/CT scans is clinically relevant in both the pre- and posttreatment settings of MM. Especially, the identification of robust positivity cut-offs for outcome prediction would have beneficial implications in the management of the disease. In this context, MTV and TLG have been proposed as promising metabolic parameters for the quantification of tumor burden and outcome prediction in MM [9, 10, 12]. At the same time, however, the accurate calculation of these parameters can be a very demanding task since it requires great computing power as well as fast and reproducible computer programs, enabling proper segmentation and correction of the background activity and partial volume effect [25]. The herein proposed approach, involving a combination of AI-based segmentation of the skeleton and subsequent thresholding of metabolic activity, aimed to objectively address these issues, enabling an automated, volumetric assessment of the BM metabolism in MM patients.

There are three major findings after the initial application of our deep learning-based tool in MM: firstly, the automated, volumetric, whole-body assessment of the intensity of BM metabolic activity in PET/CT images is feasible. Secondly, the AI-derived PET/CT biomarkers MTV and TLG are significantly correlated with the visual (subjective) analysis of the extent of BM involvement in [^18^F]FDG PET/CT images. Thirdly, automatically based MTV and TLG values are also significantly correlated with the degree of BM plasma cell infiltration rate and the independent prognostic factor β2-microglobulin after the application of certain [^18^F]FDG uptake thresholds.

The herein applied deep learning, whole body, volumetric quantification method of [^18^F]FDG metabolism in the BM is based on the initial CT-based segmentation of the skeleton, its transfer in the PET images, the application of different thresholds of tracer uptake, and the subsequent refinement of the resulting regions using postprocessing. Global thresholding for bone segmentation has only recently been applied in the setting of MM with promising results. Takahashi et al. developed a semi-automated, quantitative parameter, defined as the intensity of bone involvement (IBI), for the assessment of the amount and extent of [^18^F]FDG uptake based on SUV metrics, using liver SUV as a threshold to determine metabolically active volumes in the skeleton. After the categorization of MM patients into three groups, based on the degree of visually assessed bone involvement in PET/CT, which served as a reference, the authors found significant differences between the three groups regarding the median IBI score [25]. The same group evaluated the parameter IBI for monitoring outcomes in MM. Again, after categorization of patients into three groups based on the visual analysis of PET/CT (PET-remission, PET-stable, and PET-progression), the authors found that the IBI variation (ΔIBI) between two consecutive scans was related to the outcome in PET/CT as evaluated visually, while, moreover, significant differences in ΔIBI were found between the three groups [29]. In our study, patients were also classified into three groups based on visual and semi-quantitative evaluation of the PET/CT scans, after taking into account parameters suggested by the literature [8, 24, 25, 30]. We could, similarly, demonstrate a significant positive correlation between automatically derived PET parameters for all six thresholds and the degree of BM involvement in PET/CT as assessed by visual analysis. Moreover, significant differences were highlighted between the three patient groups regarding the MTV and TLG values for all applied thresholds. Of note is the—partly marked—variance in the yielded MTV and TLG values between the different approaches (Approaches 1–6) employed, which highlights the sensitivity of whole body calculations depending on the applied [^18^F]FDG uptake threshold, thus calling for caution in the routine use of the tool depending on the respective clinical setting.

Another distinguishing point between this work and previous ones in the field is that in our study, we went one step further and managed to show a significant moderate correlation between the AI-derived MTV and TLG and two clinically relevant biomarkers in MM. In specific, the demonstration of the correlation between the automated, volumetric PET parameters—derived by four of the evaluated approaches (Approaches 1, 2, 4, and 5)—and the percentage of BM plasma cells derived from biopsies, a main histopathological biomarker in the disease, and the levels of β2-microglobulin, a powerful predictor of survival and a key variable of ISS [31–33], significantly enhanced the robustness of our analysis, suggesting four of the applied thresholds as potentially useful cut-off values for reliable segmentation of the pathological skeleton. Moreover, the application of these four thresholds provided the best results in terms of discrimination of the studied population according to the degree of disease severity, using as a cut-off the BM plasma cell infiltration of 60% [20, 34]. These approaches were based on the comparison of [^18^F]FDG metabolism in the BM either with the tracer activity in reference organs which show very low variability and a narrow range in tracer uptake (liver and gluteal muscles) [35–37] or with absolute SUV values [12]. The reason for the partial use of different pathological uptake thresholds for the axial skeleton and the long bones is based on the fact that [^18^F]FDG uptake in the skeleton is not uniform, gradually decreasing from the axial to the appendicular skeleton [38]. Based on the present findings, these approaches will be further evaluated in future studies with larger patient cohorts. On the other hand, two of the applied thresholds (Approaches 4 and 6) failed to either demonstrate statistically significant correlations with the abovementioned clinical parameters or to discriminate the patient population based on the degree of BM plasma cell infiltration, which is attributed to the use of high [^18^F]FDG uptake thresholds, leading to rather low whole body MTV and TLG values.

The interest in volumetric PET measurements in MM is not new. Fonti et al. were the first to explore the predictive role of MTV and TLG in a mixed group of 47 MM patients who received various therapies. Their analysis was based on the identification of focal lesions and the calculation of SUV_max_. Afterwards, MTV was calculated in those lesions with a SUV_max_ > 2.5, which was almost the same as one of the thresholds applied in our analysis (SUV_max_ ≥ 2.5, Approaches 5 and 6) that led to a significant correlation between the automated MTV and TLG values and the percentage of BM plasma cells and β2-microglobulin. Similarly to our results, the authors noted that MTV positively correlated with the percentage of BM infiltration by plasma cells (*r* = 0.46), while TLG correlated significantly with β2-microglobulin levels (*r* = 0.38). They could, moreover, show that an MTV value of 77.6 mL and a TLG value of 201.4 g predicted patients with a good OS [9]. In line with this, in a larger and more homogeneous MM cohort, McDonald et al. found that baseline TLG > 620 g and total MTV > 210 mL of MM lesions were significant factors in poor PFS and OS. In that study, MM lesions were defined as foci of increased [^18^F]FDG uptake exhibiting a peak SUV (SUV_peak_) greater than that of background BM assessed in the most inferior vertebral body [10]. These findings are in agreement with the ones in the present analysis. However, an essential difference between the aforementioned studies and ours is that these approaches were not automated, and were, thus, dependent on ROI definition, which was not the case in our analysis.

Recently, in a retrospective analysis of 185 patients with newly diagnosed MM, Terao et al. investigated the predictive value of pre-treatment MTV and TLG, as assessed by a semi-automated, computer-aided analysis of the PET/CT images, and compared it with conventional PET/CT variables. The authors could show that the high-burden MTV and TLG findings were superior to the conventional high-risk PET/CT variables for outcome prediction, as assessed by PFS and OS [12]. Similarly to our results, in another study of the same group, a significant correlation between TLG and the percentage of plasma cells in the BM was demonstrated, rendering this PET parameter potentially suitable for evaluating the histopathological tumor burden in MM [39]. Notably, in the studies by Terao et al., MTV was defined as the volume of myeloma lesions with SUV ≥ 2.5, a threshold also herein applied (Approaches 5 and 6) that led to significant correlations between the PET, histopathological, and clinical parameters.

We note some limitations in our study. Foremost, the number of patients enrolled and PET/CT scans analyzed was relatively small. However, the studied cohort is homogeneous, consisting of treatment-naive, symptomatic MM patients examined in terms of an ongoing prospective study. Therefore, the presented findings can only be considered the preliminary results of an ongoing study. Secondly, the vast majority of PET/CT findings were not histopathologically confirmed, which is, obviously, not possible in the clinical setting. However, the demonstration of a significant correlation with two commonly accepted reference standards, namely, the percentage of BM infiltration by malignant plasma cells as derived from biopsies of the iliac crest and the plasma levels of β2-microglobulin, essentially contributed to the validation of the results. Moreover, especially with regard to the diffuse BM uptake pattern, in an effort to reduce the incidence of false positive findings, it was ensured that no included patient had previously received agents or medications, which could lead to a diffusely increased tracer accumulation in the BM, at least one month before the PET/CT study [40]. Furthermore, limitations exist with regard to the applied segmentation method: the calculation of MTV and TLG is SUV-dependent, meaning that every factor affecting SUV calculations may also affect the evaluation of these parameters. Moreover, the patient’s skull was excluded from the segmentation analysis and subsequent metabolic parameters’ calculation due to the very high-lying diffuse [^18^F]FDG uptake of the brain, rendering the skull as an “obscured site” [1]. Although in our sample, no patient had metabolically active, focal, cranial [^18^F]FDG-avid lesions, this anatomical area must be analyzed independently, inevitably making the method more operator-dependent in selected MM cases with cranial involvement. Finally, extensive lytic or paramedullary lesions, i.e., soft tissue/extraosseous lesions originating from bone lesions (Fig. 3), may be an additional source of error, subsequently leading to the need for manual corrections; since the AI tool initially makes a CT-based identification of the skeleton based on the HU scale of each region, it may be possible that large osteolytic lesions or soft tissue infiltrations linked to skeletal involvement are excluded from the BM segmentation. These issues will be specifically investigated in the future in a larger patient cohort in the context of this multicenter, randomized phase 3 trial, with the goal of validating the AI-based automated PET results in comparison to patient outcome data as well as the findings of whole-body MRI, which is considered the modality of choice for bone marrow evaluation and assessment of disease extent in MM patients [41].

## Conclusion

In an attempt to address the issue of the standardization of the [^18^F]FDG PET/CT interpretation in MM, we validated a novel three-dimensional deep learning-based tool on PET/CT images for automated assessment of the intensity of BM metabolism in a group of 35 consecutive, symptomatic, treatment-naive MM patients. We could show that BM segmentation and calculation of whole-body MTV and TLG after the application of the deep learning tool were feasible in all patients. Moreover, the AI-derived quantitative PET parameters correlated significantly with the results of the visual analysis of the PET/CT scans as well as with the biopsy-derived BM plasma cell infiltration and the plasma levels of β2-microglobulin. These preliminary results suggest that the automated, volumetric, whole-body PET/CT assessment of the BM metabolic activity after the application of the deep learning-based tool is a potentially reliable method in the direction of optimization and standardization of PET/CT interpretation in MM and will be further evaluated in future prospective studies with larger patient cohorts.

## Data Availability

The datasets generated during and/or analyzed during the current study are available from the corresponding author on reasonable request.
